# Supplementary material for: “Help! I need some music!”: Analysing music discourse & depression on Reddit
Source: PLoS One. 2023 Jul 20;18(7):e0287975. doi: 10.1371/journal.pone.0287975 (PMC10359011; doi:10.1371/journal.pone.0287975)
Supplement: Attachment — Submitted filename: Response_letter.pdf [file pone.0287975.s001.pdf]

Dear Reviewers,

We hereby resubmit our revised manuscript entitled "“Help! I need some music!”: Analysing Music Discourse & Depression on Reddit" to be considered for publication in PLOS ONE.

The comments by the reviewers have been extremely helpful in guiding our revision process. We agree with all the points raised by the reviewers and have restructured our manuscript accordingly.

Specifically, we have revised our methodology sections based on the comments given by the reviewers and expanded further on some of the limitations. We are now providing a more detailed description of the entire topic modelling pipeline with a separate figure explaining the different components involved.

As instructed by the reviewers, we have also made our entire dataset public now and included a statement specifying that the collection and analysis method complied with the terms and conditions for the source of the data. The dataset can be found here: https://www.kaggle.com/datasets/bhavyajeet/reddit-music-discourse

Altogether, we think that our revised manuscript has strongly benefited from the reviewers' feedback and provides an improved version.
Below we provide a detailed discussion of the reviewers' comments and our responses. Our responses are indented.

Reviewer #1

This paper - “Help! I need some music!”: Analysing music discourse & depression on Reddit - reports on work that is at the forefront of online data collection methods in this area. With that in mind, most of the comments below are provided with the intention of helping to increase the impact of the work.

With this in mind, it could be helpful to be more explicit about what each step included. For example when describing Topic Modelling, is it possible to give more information about how this worked. Perhaps an example of going through the process would help.

> In order to explain the procedures more explicitly, we have rewritten parts of the methodology section. Specifically, we have defined topic modelling in further detail with an additional diagram explaining the entire pipeline.

It might be good to illustrate what the clusters were of (words, segments, sentences) and how these were analysed thematically. That might help explain how “anxiety” is in “calm” and

why "mechanism" is presented as a separate term in "coping mechanism". Similarly, it might be helpful to provide an example of the thematic analysis in action. Finally, it might be helpful to provide quotes associated with each of the themes so that readers have an idea of the richness of the data underlying the findings.

We have explained the topic modelling section with greater clarity now (please refer to the comment above) and we believe that this would help the readers get a better idea of the entire process. Topic descriptions provide the most prevalent n-grams from the clustered sentences.
'Anxiety' is an n-gram in the "relaxation/calm" cluster due to the presence of example posts like the following in the corresponding clusters.

- "Music that ease anxiety?"
- "Relaxing/therapeutic music for depression and anxiety".
- "Overcome anxiety. Relaxing music, tested method"

We have added the following in the manuscript to clarify the procedure:
"The representative n-grams for the clusters are extracted using a statistical method, the embeddings used for clustering are contextual in nature. This allows the clustering algorithm to utilise deeper semantic meanings while grouping the posts." ….
"As described in the methodology section, the clusters are created based on contextual information whereas the representative n-grams are generated using a statistical method. This explains that while words like 'stress' or 'anxiety' from the 'topic n-grams' column of table 2 might seem contradictory to the theme of 'relaxing/calming', examining the context in which these words were used provides greater clarity. The aforementioned cluster contained posts like 'Music that ease anxiety?' or 'Relaxation music for deep sleep and stress relief' which fit well with the assigned theme."

Similarly, since 'mechanism' also appeared as a separate n-gram (without being preceded by 'coping'), it is present in the list of representative n-grams given by the model. This can be seen in the following examples :

- "Music as a mechanism to cope with life"
- "Anyone else use music as a mechanism to cope?"

Furthermore, the entire dataset has been now made public to further provide the exact quotes associated with each of the themes. This would provide the readers a better picture of the actual quotes that are associated with the identified themes in the study.

If you'd like people who are interested in the psychology of music but may not know about big data techniques, then I'd illustrate the technical process more fully. If your main audience

is other researchers working in big data, then much of this would presumably be more familiar. Connected to this, there are several technical terms which may not be familiar to readers from fields like music psychology or music and health (such as "latent semantic representations"). It may be worth how many of these you explain or at least point readers to sources where they could find out more. With that in mind, it might be worth checking throughout for readability across the different audiences you would like to engage.

We would certainly hope our work reaches a wider audience. Hence, we have restructured the methodology section to improve the clarity of our procedure. We have rewritten the procedures using simpler terminology and made sure that relevant references are provided for all technical terms and assumptions. The topic modelling procedure is now illustrated using a more detailed and understandable diagram.

The use of Reddit is an interesting idea with a lot of potential for collecting a lot of data. As the authors point out, it is not possible to know about the identity of participants. Is there any way in which readers can get a sense of who typically might use reddit (in terms of geography, age, etc.) and what that might say about what can (or can't) be learnt from the study.

We agree that there is a lack of information regarding the identity or even the demographics of the participants. However as mentioned in the paper, reddit is an anonymous platform and does not provide personal information about the users. Statistica is a popular source for obtaining statistics such as traffic on a particular website and other user demographics including age and gender distribution but the source of those numbers is unclear for Reddit. According to Statistica, the majority of users on Reddit are male and under 50 years of age with most traffic coming from western countries with USA, UK and Canada being the top 3 contributors. However these numbers only represent the users of the entire platform and not specific to the subreddits considered in the study.

We have now added the following in the Data Collection Part of the manuscript:

"While it is not possible to obtain the demographics of a particular subreddit, Statistica, a popular source for obtaining website statistics, reports that the majority of users on Reddit are male and under 50 years of age with most traffic coming from western countries with USA, UK and Canada being the top 3 contributors. However these numbers only represent the users of the entire platform and not specific to the subreddits considered in the current study."

We have also addressed this in the Limitations and Future Work section by rephrasing the following sentences:

“While user anonymity on Reddit enables users to be more forthright about their experiences on any topic, especially those associated with taboo, it does not share users’ personal information. Consequently, the research stops short of studying the relationship between music listening behaviour and individual traits, which has been done by prior studies. Individual differences such as Personality, Empathic traits, or Gender among other demographic variables may indeed be significant modulators of music listening strategies.”

The part of the title before the colon looks like it is a quote. Is this from the dataset? If so, it might be good to use it as part of one of the examples or have it featured somewhere in the text. If it isn’t from the dataset, consider modifying the title.

That part of the title has been used as a reference to the 1965 Beatles track titled “Help!”. The exact phrase does not appear in our dataset, however close variations from various contexts like “Music helps so much”, “I need music”, or “I need help with my music addiction … Please help!” do exist in our dataset.

I look forward to seeing this work in print.

We are thankful for your comments. They have helped us in improving the quality of the manuscript.

Reviewer #2

This research seeks to examine the relationship between music listening strategies and their impact on the well-being of individuals with depression. The authors argue that while music can play an important role in regulating mood and emotions, using music as a coping mechanism can lead to adverse outcomes such as anxiety and depression. They use a mixed-methods approach, including natural language processing and qualitative analysis, to identify healthy and unhealthy music-listening strategies used by individuals on Reddit. The authors also review previous studies on music engagement strategies associated with depression, the limitations of prior research, and the potential of Reddit as a data source.

The introduction is well-structured, informative, and lays a solid foundation for the study. The authors provide extensive background information and highlight the research gap, which adds significance to their study. Overall, the introduction to the paper is well-written and effectively conveys the importance of the research question. Moreover, the method they use to investigate this issue is clever and well executed — looking as it does at the music people choose to listen to on Spotify and examining the relationship between the lyrical content of this and their mood as gauged through their posts on Reddit.

Although it has some obvious limitations, I think the patterns that the study identifies are very interesting — and certainly worth reporting and reflecting on. As the authors note, some of these are to do with the gross nature of the measures. However, the one obvious limitation (that the authors don't really engage with) is that the study is correlational and hence can't tease out cause and effect (does negative affect dictate musical preferences or the other way round?). However, as the authors note the study does provide insight into the way that mood and behaviour go together and hence is a useful — and to my mind really rather interesting — contribution to the literature.

We thank Reviewer 2 for the positive comments.

We agree that our study is correlational and hence can't tease out cause and effect. In the manuscript we do not claim that this study can be used to interpret cause or effect, however we have now explicitly included this as one of the limitations in the 'limitations' sections of the paper.

This information is presented in the paper as follows:
"While this study highlights interesting patterns in the usage and sharing of music in the context of depression, we must note that these insights are correlational and not causal in nature. "
